# Improvement in Quality of Life with OnabotulinumtoxinA for Cervical Dystonia: POSTURe

**DOI:** 10.1017/cjn.2020.275

**Published:** 2021-09

**Authors:** Marc Petitclerc, Martin Cloutier, Pierre Naud, Mélanie Langlois, Meetu Bhogal, Goran Davidovic

**Affiliations:** Clinique Neuro-Lévis, Lévis, Québec, Canada; Clinique Neuro-Rive Sud, Greenfield Park, Québec, Canada; Complex Santé de la Capitale, Québec, Québec, Canada; CHU de Québec, Hôpital de l’Enfant-Jésus, Québec, Canada; Allergan plc, Markham, Ontario, Canada

**Keywords:** Cervical dystonia, OnabotulinumtoxinA, Patient-reported outcomes

## Abstract

**Introduction:**

Symptoms of cervical dystonia (CD) can vary in severity and cause significant pain. OnabotulinumtoxinA is an approved treatment for CD. This study assessed health-related quality of life (HRQoL) in patients with CD who received multiple onabotulinumtoxinA treatments.

**Methods:**

This prospective, observational standard-of-care study was conducted at multiple neurology centers in Québec, Canada. Patients reported the health impact of CD using the Cervical Dystonia Impact Profile (CDIP)-58, before and after up to eight onabotulinumtoxinA treatments. Other measures included the Cervical Dystonia Severity Rating Scale by physician, employment status using the Work Productivity Questionnaire and pain using the Pain Numeric Rating Scale (PNRS). Adverse events (AEs) were recorded.

**Results:**

Sixty-two patients were enrolled (safety population, n = 61; modified efficacy population, n = 58). Participants were mostly females who were employed; most (79.3%) had torticollis. In all, 21/62 patients (33.9%) discontinued the study. At the final visit, there was a statistically significant (*p* < 0.001) improvement in all eight CDIP-58 subscales, particularly head and neck symptoms (−31.0) and psychosocial functioning (−28.2). Employment increased from baseline (55%) to the end of the study (64%), and there was improvement in work productivity. There was a significant (*p* < 0.0001) reduction in pain measured by the PNRS, from −0.5 post-treatment 1 to −2.4 at end of study. AEs (neck pain, muscular weakness, dysphagia, nausea) were consistent with onabotulinumtoxinA use.

**Conclusion:**

These real-world data indicate that after repeated, long-term use, onabotulinumtoxinA continues to be a safe and effective treatment for CD, improving HRQoL and work productivity.

## RÉSUMÉ

L’onabotulinumtoxine A et l’amélioration de la qualité de vie des malades atteints de dystonie cervicale : POSTURe.

**Introduction:**

Les symptômes de la dystonie cervicale sont d’intensité variable et peuvent causer de la douleur importante; l’onabotulinumtoxine A est un médicament approuvé dans le traitement de ce trouble. L’étude visait à évaluer la qualité de vie liée à la santé (QVLS) chez des patients souffrant de dystonie cervicale et ayant reçu plusieurs injections d’onabotulinumtoxine A.

**Méthode:**

Il s’agit d’une étude d’observation, prospective, portant sur les soins usuels et réalisée dans plusieurs centres de neurologie, au Québec, au Canada. Les participants à l’étude ont fait état des répercussions de la dystonie cervicale sur leur qualité de vie, dans le questionnaire Cervical Dystonia Impact Profile (CDIP)58, avant et après l’administration de 1 à 8 injections au maximum d’onabotulinumtoxine A. D’autres questionnaires ont aussi été utilisés, notamment le Cervical Dystonia Severity Rating Scale, rempli par le médecin; le Work Productivity Questionnaire pour évaluer la situation de l’emploi; et le Pain Numeric Rating Scale (PNRS) pour évaluer la douleur. Ont également été consignés les effets indésirables (EI) du médicament.

**Résultats:**

Au total, 62 patients ont été retenus pour participer à l’étude (innocuité : population = 61 sujets; efficacité : population modifiée = 58 sujets). Il s’agissait en très grande partie de travailleuses; la plupart d’entre elles (79,3 %) souffraient de torticolis. Dans l’ensemble, 21 sujets sur 62 (33,9 %) se sont retirés de l’étude. À la dernière consultation, une amélioration statistiquement significative (*p*< 0,001) a été enregistrée dans les 8 sous-domaines d’évaluation du CDIP-58, notamment en ce qui concerne les symptômes affectant la tête et le cou (−31,0) et le fonctionnement psychosocial (−28,2). Une amélioration de la situation de l’emploi a été notée, du début (55 %) à la fin de l’étude (64 %), de même qu’une augmentation de la productivité au travail. D’après le questionnaire PNRS, il y a eu une réduction importante (*p*< 0,0001) de la douleur, résultat qui est passé de −0,5 après le 1^er^ traitement à −2,4 à la fin de l’étude. Enfin, les EI (douleur cervicale, faiblesse musculaire, dysphagie, nausées) étaient compatibles avec ceux associés à l’emploi de l’onabotulinumtoxine A.

**Conclusion:**

Les données factuelles recueillies révèlent que l’emploi répété, voire prolongé, de l’onabotulinumtoxine A est un traitement sûr et efficace de la dystonie cervicale, tout en permettant une amélioration de la QVLS et de la productivité au travail.

## Introduction

Dystonia is a neurological disorder that causes involuntary muscle contractions, which usually result in repetitive movements or abnormal postures or positions,^1^ and can affect a single part of the body, multiple muscles, or even the whole body.^2^ Focal dystonia involving the muscles of the neck and shoulders is termed cervical dystonia (CD) and is the most common form of dystonia; it often manifests as pulling and jerking movements of the head and neck.^3^ Sustained abnormal posture of the head and neck can be associated with fast or slow intermittent movement and tremor. The prevalence of CD has been estimated to be in the range of 20 to 4100 cases per million individuals, and it occurs more commonly in women than in men.^4^ The neck may move in several ways; in rotational torticollis, the sternocleidomastoid, splenius capitis, and obliquus capitis muscles are typically involved, whereas other more complex movements such as tilting (laterocollis) or forward (anterocollis) or backward (retrocollis) bending of the neck involve other muscle groups.^5^ The most common presentation of CD is torticollis, followed by laterocollis, retrocollis, and anterocollis, but the majority of patients experience a combination of deviations.^6^ Pain is the main reason for patients seeking treatment, with up to 90% of patients reporting pain associated with CD that greatly affects their quality of life (QoL).^7,8^ Symptoms can range from mild to severe and have significant economic impact on the patient; CD has been reported to cause loss of employment in 18.9% to 38.5% of patients.^9,10^

Botulinum toxin is a first-line treatment for CD that acts specifically at cholinergic synapses by cleaving proteins involved in vesicle fusion, and thereby prevents the release of acetylcholine at the neuromuscular junction, causing muscle relaxation.^11,12^ OnabotulinumtoxinA was the first botulinum toxin formulation approved for the treatment of CD in Canada in 1995; studies have demonstrated the benefit of treating CD with botulinum toxin type A.^13–16^ However, despite the increasing importance of patient-reported outcomes in determining whether improvements in clinical measures are being translated into meaningful functional outcomes, only a few studies have prospectively reported such measures,^16^ with most including patients who were already on stable treatment with onabotulinumtoxinA and/or only single treatments.^17–20^ Results from a Canadian long-term, multi-center, prospective observational study (MDs on BOTOX Utility; MOBILITY®) revealed that patients maintained or improved health utility scores (assessed with the Short-Form Six-Dimension Health Survey) regardless of prior treatment with onabotulinumtoxinA.^21^ Likewise, the Cervical Dystonia Patient Registry for Observation of OnabotulinumtoxinA Efficacy (CD PROBE) found significant improvements from baseline in patient-reported measures up to three treatments.^16^ However, because patients with CD are treated over the long term, it was of interest to study a greater number of treatments.

The primary objective of this study was to prospectively assess health-related QoL (HRQoL) in patients with CD over multiple treatments with onabotulinumtoxinA. Secondary objectives were to assess employability and work productivity before and after treatment with onabotulinumtoxinA; describe the initial response to onabotulinumtoxinA in terms of symptoms, daily activities, and psychosocial sequelae in patients naïve to treatment; descriptively assess treatment with onabotulinumtoxinA from the patient perspective; identify potential predictors for health and treatment outcomes, including baseline presentation and CD characteristics; and assess why patients stop treatment with onabotulinumtoxinA.

## Methods

This was a multi-center, prospective, observational standard-of-care study (ClinicalTrials.gov identifier, NCT01655862), designed to collect data on the impact of treatment with onabotulinumtoxinA on CD using patient-reported outcomes, and was designed to be minimally intrusive to both physicians and patients in order to decrease selection bias. It was conducted in multiple neurology centers within the province of Québec, Canada, in which onabotulinumtoxinA was prescribed as standard of care; data from all centers were pooled. OnabotulinumtoxinA was administered as deemed appropriate by the treating physician. The study period included 10 study assessments comprising two in-office physician assessments (initial [baseline] and final [prior to the ninth injection] visits) and eight telephone assessments. There were also up to eight in-office patient treatment visits.

### Patient Eligibility

To be included in the study, patients with CD had to be eligible to receive botulinum toxin type A treatment as deemed medically necessary by the participating physician independently from this project. Patients were male or female and at least 16 years of age on the day of informed consent. Patients concurrently participating in a clinical trial for any botulinum toxin indication; those with any contraindications to use of any botulinum toxin according to the approved product information or who had any condition or situation which, in the physician’s opinion, placed the patient at significant risk, could confound the study data or interfere significantly with the patient’s participation in the study, including but not limited to unstable medical conditions; those with planned elective surgery during the observational study period; and those with a history of poor cooperation or non-compliance with medical treatment were excluded from study entry. Females who were pregnant, nursing, or planning a pregnancy during the study period were excluded. Patients who had received treatment with any botulinum toxin product for CD or for a non-CD condition within 2 months of study start were also ineligible for study entry.

### Assessments

Patients reported the health impact of CD before (at baseline) and after treatment with onabotulinumtoxinA (at 4 weeks after treatments 1, 3, 5, and 7 and at the final study visit) using the Cervical Dystonia Impact Profile (CDIP)-58, a validated, patient-based, disease-specific questionnaire that measures QoL in patients with CD.^18^ The instrument is made up of 58 items forming eight subscales: head and neck symptoms, pain and discomfort symptoms, upper limb activities, walking, sleep, annoyance, mood, and psychosocial functioning. The total was a transformed score ranging from 0 to 100, with 0 indicating best possible QoL and 100 indicating the worst possible QoL. The Cervical Dystonia Severity Rating Scale is a subjective physician assessment used to compare the patient’s CD against the most severe case that physician has seen in their practice according to mild, moderate, or severe; physicians completed this at baseline. Employment status, the effect of CD on employment and the effect of medications, including side effects, on work productivity were assessed using the Work Productivity Questionnaire (WPQ), a multi-item instrument that assesses work productivity over the previous 7 days.^9^ The WPQ was administered at the initial baseline, 8 weeks after treatments 1, 3, 5, and 7, and at the final study visit. Patients scored the amount of pain they experienced before (at baseline) and after treatment (at 4 weeks after treatments 1, 3, 5, and 7 and at the final study visit) using the Pain Numeric Rating Scale (PNRS), a single-item questionnaire that asks the patient to respond to the following question: “Please rate the pain you have experienced during the last 24 hours on a scale from 0 to 10” where 0 indicates “no pain” and 10 indicates “pain as bad as you can imagine”. Patients were asked to complete the Hospital Anxiety and Depression (HAD) questionnaire at baseline and the final study visit. The HAD scale is a 14-item instrument used to assess depression and anxiety of patients in which patients are asked to “Place an ‘X’ on the answer that best describes how you have been feeling during the last week”. The answer can range from 0 (not at all/only occasionally) to 3 (definitely/often). Both patients and physicians rated the severity of the patient’s illness using the Global Impression of Change, in which a question was answered on a seven-point scale, from 1 (very much improved) to 7 (very much worse). For the Patient’s Global Impression of Change (PGIC; administered at 8 weeks after treatments 1, 3, 5, and 7 and at the final study visit), the patient was asked to answer the following question: “Compared to your condition at admission to this study, please rate your total change whether or not, in your judgment, it is due entirely to drug treatment”. For the Clinician’s Global Impression of Change (CGIC; administered at the final study visit), the clinician was asked to answer the following question: “Compared to the patient’s condition at admission to this study, how much has he or she changed?” Time to symptom re-emergence was used to assess when treatment began to diminish and whether the patient wanted to receive another onabotulinumtoxinA treatment after treatments 2 to 8 and at the final study visit. Adverse events (AEs) were recorded throughout the entire study duration.

### Schedule of Assessments

At the baseline visit (first onabotulinumtoxinA treatment), informed/patient consent and demographic, medical history, and current status information were obtained. Classification of CD was performed; physician- (Cervical Dystonia Severity Rating Scale) and patient-reported outcomes (CDIP-58 questionnaire, WPQ, PNRS, and HAD scale) were completed before treatment with onabotulinumtoxinA. The onabotulinumtoxinA injection regimen (dose, dilution, muscles injected, number of injection sites, and guidance technique used) was also determined by the treating physician who were all experienced injectors. Incidence of dysphagia at baseline was not recorded in this study. Treatment visits occurred on the day of retreatment with onabotulinumtoxinA. The following procedures were performed: onabotulinumtoxinA injection regimen; Physician’s Treatment Satisfaction questionnaire; time to symptom re-emergence (completed by the patient); concomitant medications; and AEs.

At the first telephone assessment, at 4 weeks (±7 days after the first, third, fifth and seventh injections), CDIP-58, PNRS, and concomitant medications were assessed. At the second telephone assessment, at 8 weeks (±7 days after the first, third, fifth, and seventh injections), the WPQ, PGIC, and concomitant medications were assessed. At the final visit (before the ninth injection) medical history review, CD classification, CGIC, Cervical Dystonia Severity Rating Scale, Physician’s Treatment Satisfaction questionnaire, WPQ, CDIP-58, PNRS, PGIC, HAD scale, time to symptom re-emergence, concomitant medications, onabotulinumtoxinA injection regimen (dose, dilution, muscles injected, number of injection sites, and guidance technique used), and AEs were assessed.

### Statistical Analysis

This study was exploratory, and as such, no formal sample size calculations were performed. The “all patients population” included all patients who provided a signed and dated informed consent and were entered into the electronic data capture system. This population was used to provide descriptive summaries for patients enrolled by center, patient disposition, and reasons for screen failure. The “safety population” included patients from the “all patients population” who had received at least one injection of onabotulinumtoxinA. The “modified efficacy population” included patients from the “all patients population” and who had a confirmed diagnosis of CD and had received at least one injection of onabotulinumtoxinA. The primary and secondary analyses were performed using the modified efficacy population. The safety and treatment analyses were performed using the safety population. Treatment-emergent AE (TEAE) data were tabulated by system organ class and preferred term utilizing the Medical Dictionary for Regulatory Activities (MedDRA™ version 19.1).

## Results

### Patient Disposition

Between July 20, 2012 and February 17, 2017, a total of 62 patients (all-patient population) were enrolled from eight centers in Canada (Clinic Neuro-Lévis QC, n = 17; Clinique Neuro Rive-Sud QC, n = 15; Montreal Neurological Institute-Hospital QC, n = 3; Hôpital Fleurimont (CHUS) QC, n = 2; CHU de Québec, Hôpital de l’Enfant-Jésus QC, n = 5; Hôpital Général Juif Montreal, QC, n = 7; CHUM Centre Hospitalier Universitaire de Montreal, QC, n = 7; Complex Santé de la Capitale Quebec City, QC, n = 6) and provided informed consent and were entered into the electronic data capture system. Of these, 61 patients received at least one injection of onabotulinumtoxinA and comprised the “safety population”; 58 patients had a confirmed diagnosis of CD and received at least one injection of onabotulinumtoxinA and comprised the “modified efficacy population”. A total of 21 of the overall 62 patients (33.9%) discontinued the study; one patient (1.6%) did not meet the inclusion/exclusion criteria; two (3.2%) were lost to follow-up; three (4.8%) discontinued because of an AE or serious AE (SAE), and 15 (24.2%) owing to other reasons. Of 17 patients who were asked, 15 completed a withdrawal questionnaire; reasons included pain at injection site and side effects of treatment (both reported by the same patient); pregnancy; treatment did not work (each n = 1), and “other”, which included lack of staffing resources at site (n = 7), lost to follow-up (n = 2), SAE (n = 1), final visit overlooked (n = 1), and patient finished study at treatment 8 rather than visit 9 per protocol (n = 1).

### Baseline Demographics and Disease Characteristics

Baseline demographics and disease characteristics are shown in Table 1. The study population comprised mostly females who were employed. A total of 54 patients (93.1%) had comorbidities prior to entering the study; the majority were musculoskeletal (57.4%), neurological (other than CD, 50.0%), cardiovascular (44.4%), and endocrine (42.6%) (Supplemental Table 1). For those patients reporting prior neurological disease (other than CD), the disease/disorder history was varied. Many of these neurological disorders would not be unexpected in the patient population studied here, and the most frequent (≥2) included essential tremor (n = 7), anxiety (n = 6), Bell’s palsy (n = 3), cerebral palsy, hand tremor, headache, head tremor, insomnia, meningioma, migraine, and vertigo (all n = 2). Seventeen patients (35.4%) had a change in medical status; most changes were in cardiovascular, gastrointestinal, musculoskeletal (all 41.2%), and neurological (other than CD, 35.3%) conditions (Supplemental Table 1). There were delays in diagnosis (8.2 years), in receiving a first treatment other than onabotulinumtoxinA (5.5 years), and in receiving first onabotulinumtoxinA treatment (10.2 years) (Table 1). Most patients had torticollis (79.3%), right-pull (62.1%); two-thirds (66.1%) had tonic contraction, 73.2% had head tremor, and 91.4% had mild or moderate disease severity (Table 1).

**Table 1:** Patient demographics and baseline characteristics

Parameter	Modified efficacy population (n = 58, unless otherwise indicated)
Age, years, mean (SD)	57.4 (11.1)
Gender, female, n (%)	44 (75.9)
Race, Caucasian, n (%)	58 (100.0)
Height,* cm, mean (SD)	163.2 (9.3)
Weight, kg, mean (SD)	72.8 (19.1)
Employment status, n (%)	
Retired	25 (43.1)
Full time	18 (31.0)
On sick leave	1 (1.7)
Part time	4 (6.9)
Unemployed	7 (12.1)
Other	3 (5.2)
Comorbidity prior to study entry, n (%)	54 (93.1)
Age at time of first symptoms,** years, mean (SD)	47.9 (12.0)
Time from first symptoms to diagnosis,** years (SD)	8.2 (8.0)
Time from first symptoms to first treatment other than onabotulinumtoxinA,*** years, mean (SD)	5.5 (7.8)
Time from first symptoms to first onabotulinumtoxinA injection treatment,** years, mean (SD)	10.2 (8.8)
Type of cervical dystonia, n (%)	
Anterocollis	3 (5.2)
Laterocollis	25 (43.1)
Retrocollis	1 (1.7)
Torticollis	46 (79.3)
Mixed	6 (10.3)
Predominant direction of pull, n (%)	
Left	22 (37.9)
Right	36 (62.1)
Characteristic of dystonic contraction,****n (%)	
Tonic	37 (66.1)
Clonic	19 (33.9)
Head tremor,**** yes, n (%)	41 (73.2)
Cervical dystonia severity rating, n (%)	
Mild	24 (41.4)
Moderate	29 (50.0)
Severe	5 (8.6)

SD = standard deviation.

*n = 57.

**n = 55.

***n = 20.

****n = 56.

### OnabotulinumtoxinA Utilization

Patients received injections over five to seven sites in an average of three muscles using a 2:1 dilution of onabotulinumtoxinA (Table 2). The mean total dose injected over treatments 1–8 was 186.9 units, which ranged from 155.7 U (at treatment 1) to 199.8 U (at treatment 4). A stable dose of approximately 190 U was reached at treatment 3 (Table 2). Across all treatments, the splenius capitis was the most targeted muscle (100% of patients), followed by the sternocleidomastoid (80.3% of patients), trapezius (59.0% of patients), and levator scapulae (44.3% of patients). The least commonly injected muscles (<10% of patients) were the scalenes, splenius cervicis, and platysma. Injection guidance was used for 11% to 21% of treatments.

**Table 2:** OnabotulinumtoxinA utilization (safety population)

					Treatment				
Mean (SD)	1 (n = 61)	2 (n = 57)	3 (n = 56)	4 (n = 55)	5 (n = 49)	6 (n = 43)	7 (n = 43)	8 (n = 43)	All (1−8) (n = 407)
Total no. of injection sites	5.5 (2.9)	6.2 (3.5)	6.9 (3.9)	7.0 (4.2)	6.7 (4.2)	6.3 (3.7)	6.5 (4.0)	6.3 (3.6)	–
Total no. of muscles injected	2.7 (1.1)	2.8 (1.0)	3.0 (1.2)	3.0 (1.2)	3.0 (1.3)	3.0 (1.2)	3.0 (1.2)	3.0 (1.1)	–
Total volume (ml)	1.9 (1.0)	2.2 (1.3)	2.4 (1.6)	2.5 (1.7)	2.3 (1.6)	2.1 (1.1)	2.1 (1.1)	2.1 (1.1)	2.2 (1.4)
Total dose (units)	155.7 (49.6)	176.4 (60.5)	191.4 (71.2)	199.8 (80.6)	196.8 (79.6)	193.0 (66.3)	197.0 (69.6)	194.9 (69.2)	186.9 (69.6)
Dose per muscle (units)									
Levator scapulae									
n (not injected)	22 (39)	18 (39)	19 (37)	19 (36)	17 (32)	17 (26)	18 (25)	17 (26)	147 (0)
Dose	41.1 (13.4)	46.1 (18.1)	45.3 (14.3)	45.8 (15.9)	49.4 (20.0)	47.6 (19.9)	47.2 (18.5)	51.8 (22.2)	46.6 (17.6)
Longissimus									
n (not injected)	1 (60)	4 (53)	6 (50)	5 (50)	6 (43)	4 (39)	4 (39)	4 (39)	34 (0)
Dose	50.0 (NA)	18.8 (6.3)	18.3 (5.2)	17.0 (4.5)	17.5 (4.2)	26.3 (16.0)	26.3 (16.0)	26.3 (16.0)	21.8 (11.1)
Scalenes									
n (not injected)	1 (60)	0 (57)	0 (56)	0 (55)	1 (48)	1 (42)	2 (41)	2 (41)	7 (0)
Dose	50.0 (NA)	–	–	–	25.0 (NA)	70.0 (NA)	60.0 (14.1)	60.0 (14.1)	55.0 (16.6)
Semispinalis									
n (not injected)	9 (52)	8 (49)	10 (46)	10 (45)	7 (42)	7 (36)	7 (36)	7 (36)	65 (0)
Dose	41.7 (20.6)	40.0 (14.1)	50.5 (29.2)	40.0 (19.6)	42.9 (28.0)	42.1 (29.1)	45.7 (26.4)	40.7 (28.9)	43.1 (23.6)
Splenius capitis									
n (not injected)	61 (0)	57 (0)	56 (0)	55 (0)	49 (0)	43 (0)	43 (0)	43 (0)	407 (0)
Dose	74.9 (27.2)	86.5 (39.2)	93.5 (48.5)	98.3 (54.1)	94.9 (55.3)	89.8 (42.0)	92.0 (44.1)	91.9 (42.7)	89.8 (44.9)
Sternocleidomastoid									
n (not injected)	45 (16)	43 (14)	45 (11)	44 (11)	39 (10)	34 (9)	34 (9)	32 (11)	316 (0)
Dose	50.9 (14.1)	51.9 (19.4)	49.1 (22.6)	51.0 (23.8)	54.0 (24.4)	55.4 (23.0)	54.6 (23.6)	55.2 (23.8)	52.5 (21.7)
Trapezius									
n (not injected)	25 (36)	27 (30)	31 (25)	29 (26)	25 (24)	22 (21)	21 (22)	22 (21)	202 (0)
Dose	50.2 (22.3)	61.9 (24.1)	54.8 (23.7)	66.7 (29.0)	62.8 (30.3)	58.0 (21.3)	60.2 (20.3)	58.0 (21.3)	59.1 (24.6)
Platysma									
n (not injected)	0 (61)	0 (57)	1 (55)	2 (53)	2 (47)	0 (43)	0 (43)	0 (43)	5 (0)
Dose	–	–	25.0 (NA)	25.0 (0.0)	25.0 (0.0)	–	–	–	25.0 (0.0)
Splenius cervicis									
n (not injected)	0 (61)	0 (57)	1 (55)	0 (55)	0 (49)	0 (43)	0 (43)	0 (43)	1 (60)
Dose	–	–	75.0 (NA)	–	–	–	–	–	75.0 (NA)

NA = not applicable; SD = standard deviation.

Across all treatment cycles, the mean (standard deviation) time between injections was 13.0 (2.0) weeks. Injection interval was consistent following each treatment (range: 12.7 [1.8] to 13.3 [2.3]; Supplemental Table 2)

### Patient-reported Outcomes

**Figure 1:**
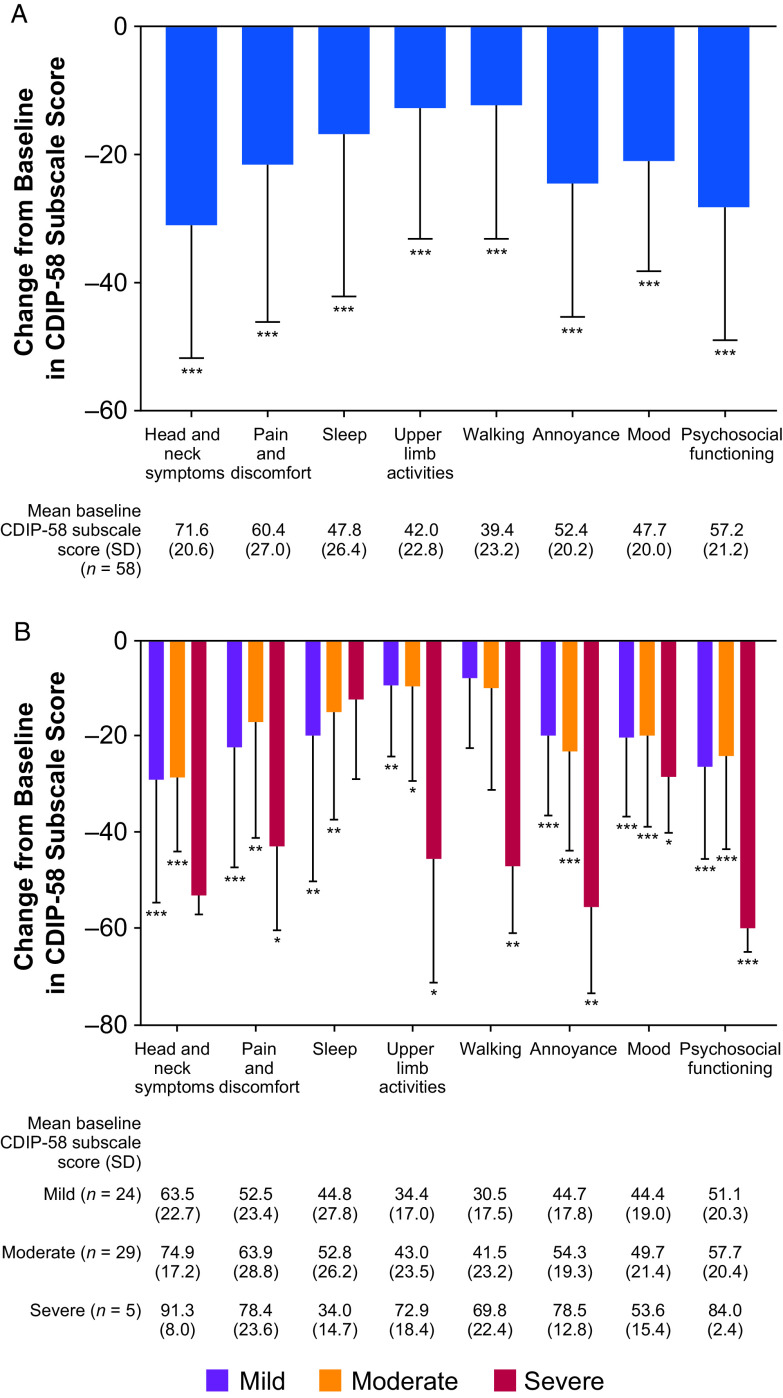
(A) Change from baseline in CDIP-58 subscales – total population; (B) CDIP-58 subscales – by CD severity rating. CDIP-58 scores determined at the final study visit. Data are mean ± standard deviation. *p < 0.05; **p < 0.01; ***p < 0.001 vs. baseline. CDIP-58 = Cervical Dystonia Impact Profile.

**Figure 2:**
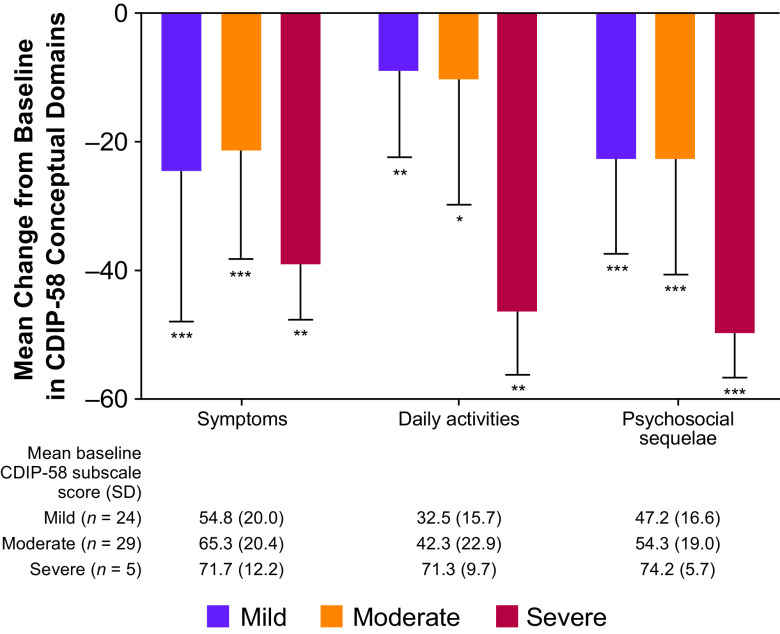
CDIP-58 conceptual domains – CD severity rating. *p < 0.01; **p < 0.05; ***p < 0.001 vs. baseline. CDIP-58 scores determined at final visit. Error bars represent standard deviation. CD = cervical dystonia; CDIP-58 = Cervical Dystonia Impact Profile.

At the final visit, there was a statistically significant improvement in all eight subscales of the CDIP-58 for the total population, particularly in head and neck, annoyance, and psychosocial functioning (Figure 1A). There was a statistically significant improvement in the majority of CDIP-58 subscales according to Cervical Dystonia Severity Rating, with the exception of head and neck symptoms and sleep in patients with severe disease, and walking in patients with mild or moderate disease (Figure 1B). There were statistically significant (*p* < 0.001) improvements from baseline in all CDIP-58 conceptual domains in the total population (symptoms, −24.1 [baseline, 61.5]; daily activities −12.6 [baseline, 40.7]; and psychosocial sequelae −25.0 [baseline, 52.7]). Improvements from baseline in CDIP-58 conceptual domains according to Cervical Dystonia Severity Rating were also statistically significant (Figure 2).

### Effect of Treatment on Employment, Work Productivity, and Health

**Figure 3:**
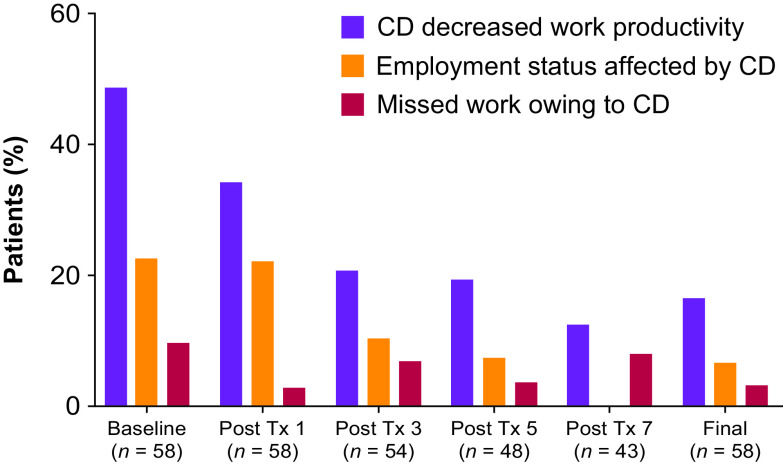
*Change from baseline in work productivity. CD = cervical dystonia; Tx = treatment*.

At the baseline visit, 32 of 58 patients (55.2%) indicated that they were currently employed. Overall, 43 of 57 patients (75.4%) stated that they were employed when their CD symptoms began, and 9 of these 43 patients (20.9%) reported that CD symptoms had caused them to stop working. Following onabotulinumtoxinA treatment, fewer employed patients with CD reported loss of work productivity than at baseline (Figure 3). There was a significant reduction from baseline over time in pain as measured by the PNRS, from −0.5 after treatment 1 to −2.4 (*p* < 0.0001) at the end of study; the largest decrease was observed at treatment 7 (4 weeks post-treatment; −2.5 ± 3.0; *p* < 0.0001). Similarly, at the final visit, there was a statistically significant decrease from baseline in anxiety (−2.3, baseline 7.1; *p* < 0.001) and depression (−1.4, baseline 4.2; *p* < 0.01) as measured by the HAD scale. On the PGIC, 76.8% of patients rated their CD as “Improved” (very much improved, much improved, minimally improved) after treatment 1 (8 weeks post-treatment); this increased to over 90% after treatments 3, 5, 7 (8 weeks post-treatment) and at the final study visit (91.5%). At each treatment, most (>70%) of the study physicians were either very satisfied or mostly satisfied with their patient’s response to previous treatment with onabotulinumtoxinA. At the final study visit, this value increased to >90% of physicians. All physicians reported on the CGIC that patients had improved. In all, 76.1% and 83.3% of patients felt that their symptoms had re-emerged after the first and second onabotulinumtoxinA treatments, respectively; this proportion decreased with subsequent treatments (before treatment 4, 70.6%; 5, 76.5%; 6, 69.7%; 7, 63.2%; 8, 61.9%; 9, 46.8%). Of those who completed the symptom re-emergence questionnaire (Supplemental Figure 1), a total of 74.3% patients reported the re-emergence of symptoms 1 to 3 weeks prior to retreatment 2. Aside from following the first treatment, the majority of patients indicated that they would prefer retreatment at 12 weeks or later (treatment 2, 45.5%; 3, 51.6%; 4, 64.5%; 5, 64.6%; 6, 72.7%; 7, 65.9%; 8, 71.5%; 9, 68.1%) and the most common interval requested was 12 weeks (36.4% to 63.6%).

### Safety

The mean duration of exposure to onabotulinumtoxinA was 16.9 months (range: 0.03–25.0 months). One hundred twenty-six AEs were reported. Of these, 121 were TEAEs, which were reported by 41 (67.2%) subjects; one was deemed to be a SAE (lung neoplasm). TEAEs occurring in more than one patient are reported in Table 3. Sixty-two TEAEs reported by 27 (44.3%) subjects were considered treatment-related. The most common treatment-related TEAEs were neck pain (17 events reported by 11 [18.0%] subjects), muscular weakness (12 events reported by 10 [16.4%] subjects), and dysphagia (13 events reported by eight [13.1%] subjects). One episode of dysphagia was classified as moderate in intensity, and the remaining 12 were mild; for 3 of the 13 episodes, a change in the treatment regimen for onabotulinumtoxinA was initiated by the investigator. All resolved without additional treatment. Adjudication by an independent safety committee of each episode of dysphagia determined that these events did not represent a distant spread of toxin.

**Table 3:** Treatment-emergent adverse events (TEAEs) (safety population)

TEAEs in > 1 patient (n = 61)	Patients, n (%)	Events, n
Neck pain	12 (19.7)	19
Muscular weakness	10 (16.4)	12
Dysphagia	8 (13.1)	13
Headache	4 (6.6)	5
Nausea	4 (6.6)	4
Dizziness	3 (4.9)	3
Vertigo	3 (4.9)	3
Dizziness postural	2 (3.3)	3
Hypoesthesia	2 (3.3)	3
Musculoskeletal pain	2 (3.3)	3
Fatigue	2 (3.3)	2
Injection site pain	2 (3.3)	2
Myalgia	2 (3.3)	2
Vertigo positional	2 (3.3)	2

## Discussion

This multi-center, prospective, observational standard-of-care study evaluated the impact of treatment with onabotulinumtoxinA on CD using patient-reported outcomes. Statistically significant improvements in CD symptoms were reported on all eight CDIP-58 subscales, and on all three CDIP-58 conceptual domains, improvements were consistent across all disease severities (mild, moderate, and severe). These improvements were coupled with statistically significant improvements in pain (as measured by the PNRS), and in anxiety and depression (as measured by the HAD scale). After treatment 3 and through the remainder of the study, over 90% of patients rated their CD as “Improved” on the PGIC, compared with 100% of physicians on the CGIC, highlighting a slight discrepancy between physician and patient impressions. Employment increased from 55% at baseline to 64% at end of study and following treatment with onabotulinumtoxinA, fewer patients reported that CD decreased work productivity (16.7% compared with 48.4% at baseline).

In the current study, patient-reported outcomes based on Physician assessment (e.g., CDIP-58) demonstrated a consistent, sustained benefit for those receiving long-term treatment with onabotulinumtoxinA, regardless of the patient’s desire to be retreated earlier based on symptom re-emergence. Approximately three quarters of patients felt that their symptoms had re-emerged after the first treatment with onabotulinumtoxinA; although this is in agreement with observations in clinical practice, this patient-reported outcome is subjective and is not physician-evaluated. The majority of patients indicated that they would prefer retreatment with onabotulinumtoxinA at 12 weeks or later. Thus, onabotulinumtoxinA administered to patients at 3-month intervals would, in most cases, be expected to provide continued benefit. This is consistent with a study of incobotulinumtoxinA, where the majority of patients (52.9%) requested retreatment later than 12 weeks.^22^ In a study of abobotulinumtoxinA, approximately half of the patients receiving lower study doses requested retreatment at 8 weeks compared with 30% of patients receiving the higher dose.^23^

In this study, patients received injections in an average of three muscles. This is similar to the CD PROBE study, in which the majority (83.2%) of patients received injections into 3 to 5 muscles.^16^ The results reported here are consistent with those seen in CD PROBE. Significant changes from baseline in each of the CDIP-58 subscale scores were observed across the three onabotulinumtoxinA treatments in CD PROBE, as were similar improvements in the PGIC and CGIC.^16^ Patients in both the present study and CD PROBE experienced similar improvements in pain as measured on the PNRS^24^ and work productivity with multiple onabotulinumtoxinA treatments.^10^ Taken together, data from both studies show that patients continue to derive benefit from onabotulinumtoxinA treatment for their CD over time and that symptom re-emergence could be used to better guide treatment goals.

OnabotulinumtoxinA was well-tolerated during this study with a safety profile similar to that observed in clinical practice. The TEAEs in this study (neck pain, muscular weakness, dysphagia, headache, and nausea) are as expected with this treatment; weakness in cervical musculature and dysphagia are the most common side effects observed with botulinum toxin treatment.^15,16^ The incidence of dysphagia (13%) was similar to that reported in clinical practice, which ranges from 3.4%^25^ to 19%,^26^ with a mean reported rate of 8.9%.^27,28^ However, patients with CD often do not report dysphagia, so it is often underdiagnosed. As dysphagia is commonly experienced by patients with CD, it is important for the physician to assess for the presence of dysphagia at baseline. In a study that measured dysphagia in 18 consecutive patients with spasmodic torticollis before and after their first treatment with onabotulinumtoxinA, pre-treatment, 11% of the patients had clinical symptoms of dysphagia and 22% had radiologic signs of a peristaltic abnormality.^29^

Typically, in studies that use questionnaires, owing to time required to complete these questionnaires, patient enrollment tends to be biased toward those who are employed part-time, unemployed, or retired. This study was designed to be minimally intrusive to both physicians and patients in order to reduce this type of selection bias.

In conclusion, these real-world data indicate that after repeated, long-term use, onabotulinumtoxinA continues to be a safe and effective treatment for CD, improving HRQoL and work productivity.

## Supplementary material

For supplementary material accompanying this paper visit http://dx.doi.org/10.1017/cjn.2020.275. click here to view supplementary material
