# Supplementary material for: Improvement in Quality of Life with OnabotulinumtoxinA for Cervical Dystonia: POSTURe
Source: Can J Neurol Sci. 2020 Dec 21;48(5):676–84. doi: 10.1017/cjn.2020.275 (PMC8527835; doi:10.1017/cjn.2020.275)
Supplement: Supplementary file 1 [file S0317167120002759sup.zip › S0317167120002759sup001.pdf]

Supplemental Table 2: Treatment Intervals

	**1 to 2 (*n* = 57)**	**2 to 3 (*n* = 56)**	**3 to 4 (*n* = 55)**	**4 to 5 (*n* = 49)**	**5 to 6 (*n* = 43)**	**6 to 7 (*n* = 43)**	**7 to 8 (*n* = 43)**	**All intervals (*N* = 346)**
Mean (SD)	13.3 (2.3)	12.7 (1.8)	13.0 (2.0)	12.9 (2.0)	13.2 (1.8)	12.8 (1.9)	12.8 (2.3)	13.0 (2.0)
Median	13.0	12.1	13.0	13.0	13.1	13.0	13.0	13.0
Min, max	8.6, 22.6	7.0, 17.9	9.7, 18.1	8.0, 18.0	10.0, 17.0	8.1, 16.0	7.6, 20.0	7.0, 22.6

SD, =standard deviation.
