# Supplementary material for: Improvement in Quality of Life with OnabotulinumtoxinA for Cervical Dystonia: POSTURe
Source: Can J Neurol Sci. 2020 Dec 21;48(5):676–84. doi: 10.1017/cjn.2020.275 (PMC8527835; doi:10.1017/cjn.2020.275)
Supplement: Supplementary file 1 [file S0317167120002759sup.zip › S0317167120002759sup002.pdf]

Supplemental Table 1: Co-morbidities prior to study entry

	**Overall (*N* = 58)**
Significant disease/disorder prior to study entry, *n* (%)	
Yes	54 (93.1)
If yes, body system with medical condition,* *n* (%)	
Musculoskeletal	31 (57.4)
Neurological (other than cervical dystonia)	27 (50.0)
Cardiovascular	24 (44.4)
Endocrine	23 (42.6)
Gastrointestinal	20 (37.0)
Psychiatric	17 (31.5)
Respiratory	9 (16.7)
Other	7 (13.0)
Genitourinary	5 (9.3)
Dermatological	3 (5.6)
Hematological	3 (5.6)
Lymphatic	3 (5.6)
HEENT	2 (3.7)
Immunological	2 (3.7)
Change in medical status,** *n* (%)	
Yes	17 (35.4)
If Yes, body system with medical condition,* *n* (%)	
Cardiovascular	7 (41.2)
Gastrointestinal	7 (41.2)
Musculoskeletal	7 (41.2)
Neurological (other than cervical dystonia)	6 (35.3)
Endocrine	3 (17.6)
Psychiatric	2 (11.8)
HEENT	1 (5.9)
Other	1 (5.9)
Respiratory	1 (5.9)

* Patients experiencing multiple comorbidities within a body system were counted only once.
** *n* = 48.
HEENT, head, eyes, ears, nose and throat.
