# Supplementary material for: Improvement in Quality of Life with OnabotulinumtoxinA for Cervical Dystonia: POSTURe
Source: Can J Neurol Sci. 2020 Dec 21;48(5):676–84. doi: 10.1017/cjn.2020.275 (PMC8527835; doi:10.1017/cjn.2020.275)
Supplement: Supplementary file 1 [file S0317167120002759sup.zip › S0317167120002759sup003.pdf]

Supplemental Figure 1: Time to waning effect of treatment or symptom reemergence questionnaire

1 – Referring to your current state of cervical dystonia, do you feel that the effects of BOTOX® for injection have started to wane?

____Yes ____No

If yes, what is the symptom that tells you that your treatment with BOTOX® for injection is wearing off? __________

If yes, when did the effects of BOTOX® for injection start to wane (circle the closest range):

1 week 2 weeks 3 weeks 4 weeks more than 1 month ago.

2 – From your perspective, at which frequency would you like to receive your BOTOX® for injection treatments? (Choose one/Circle the closest number)

Once every:
8 weeks, 9 10 11 12 13 14 15 16 >16 wks
